# Trigeminal Somatosensory Evoked Potentials During Neurosurgical Procedures Using Electrical Stimulation of the Lips Under General Anesthesia

**DOI:** 10.7759/cureus.37601

**Published:** 2023-04-15

**Authors:** Bujji Karre, Gopalakrishnan M Sasidharan, Prasanna U Bidkar

**Affiliations:** 1 Neurosurgery, Intraoperative Neurophysiology Monitoring, Jawaharlal Institute of Postgraduate Medical Education & Research, Puducherry, IND; 2 Neurosurgery, Jawaharlal Institute of Postgraduate Medical Education & Research, Puducherry, IND; 3 Anaesthesiology and Critical Care, Jawaharlal Institute of Postgraduate Medical Education & Research, Puducherry, IND

**Keywords:** neurosurgery, notch filter, total intravenous anesthesia, trigeminal sensory evoked potential, intraoperative neurophysiology

## Abstract

Background

Intraoperative neurophysiology monitoring is rapidly evolving with the advent of newer modalities. Long latency sensory evoked potentials from the trigeminal nerve distribution have rarely been demonstrated during neurosurgical procedures. Trigeminal sensory evoked potential (TSEP) can be used to prevent nerve injury during surgical procedures, such as those for trigeminal neuralgia and tumors involving the trigeminal nerve and pathway.

Methodology

We attempted to record TSEP from 12 subjects who underwent various neurosurgical procedures with low doses of inhalational anesthetic agents. We stimulated the upper and lower lip and recorded from C6 and Fz locations. We used 14-17 mA current stimuli with a pulse width of 50-150 microseconds and a stimulation rate of 2.1 Hz.

Results

We could obtain a clear, reproducible TSEP response in two out of 12 subjects. We observed a TSEP waveform with negative peaks at 13 and 27 milliseconds and a positive wave at around 19 milliseconds.

Conclusions

The TSEP produced by the electrical stimulation of the upper and lower lip can be detected from the scalp C5, C6, and Fz area even during neurosurgical procedures, even if inhalational anesthesia was used at induction, but only in a small proportion of cases. It appeared to reflect the activity of trigeminal cortical response. Avoiding the notch filter and turning off the inhalational agents are essential for a good response.

## Introduction

Intraoperative neurophysiology monitoring (IONM) is a rapidly evolving field in neurosurgery, with various techniques used to monitor sensory-motor, visual, auditory, and cranial nerves [1-4]. Median sensory evoked potentials (SSEP) are a well-established technique for studying sensory pathways [5]. Trigeminal sensory evoked potentials (TSEPs) are a sensory response of the trigeminal nerve that can be used for diagnosis or treatment evaluation in cases where the trigeminal nerve is at risk of injury, particularly in oral and maxillofacial surgeries, facial skeleton osteotomies, maxillofacial trauma, mid-face fractures, trigeminal neuralgia, and ischemic brainstem lesions involving the trigeminal nerve or in the surgery for trigeminal neurinomas [6,7].

Although TSEPs have been studied for decades, there is still controversy surrounding the source of the response [8]. Furthermore, although TSEPs have been studied in awake patients, intraoperative monitoring of TSEPs has been done rarely [6,7]. In this study, we aimed to record TSEPs during neurosurgical procedures using electrical stimulation to clarify the shape of the TSEP waveform.

## Materials and methods

After obtaining informed consent, we included 12 subjects in the study as part of a larger study to elicit olfactory evoked potentials during neurosurgery. Because olfactory mucosa also contains trigeminal sensory nerves, we needed to distinguish a trigeminal evoked response from the more elusive olfactory evoked potentials. We recruited eight females and four males, aged 27 to 64 years, all without olfactory or facial sensory disturbances, for this study after obtaining ethical clearance from Jawaharlal Institute of Postgraduate Medical Education & Research, the Institutional Ethics Committee (Human Studies) for Intervention Studies (JIP/IEC/20l9/l85).

We used low-dose inhalational agents (sevoflurane or desflurane, ranging from 0.2 to 0.3 minimum inhibitory concentration) at induction and positioning in all patients but either turned it off completely (the last but one patient) or continued at very low doses during monitoring in most patients. In addition, we used a continuous infusion of propofol and fentanyl and/or dexmedetomidine along with a muscle relaxant. We avoided muscle relaxants if we needed to monitor motor-evoked potential or cranial nerve electromyogram. We did not strictly define the anesthesia protocol in this study, but the anesthesia team was free to use the combination of drugs necessary for each case.

We recorded the brain surface temperature using an infrared thermometer, which remained at 27-30°C, and we usually maintained the body’s core temperature at 36.5°C with convective warmers. We maintained the bispectral index of 40 to 50.

**Figure 1:**
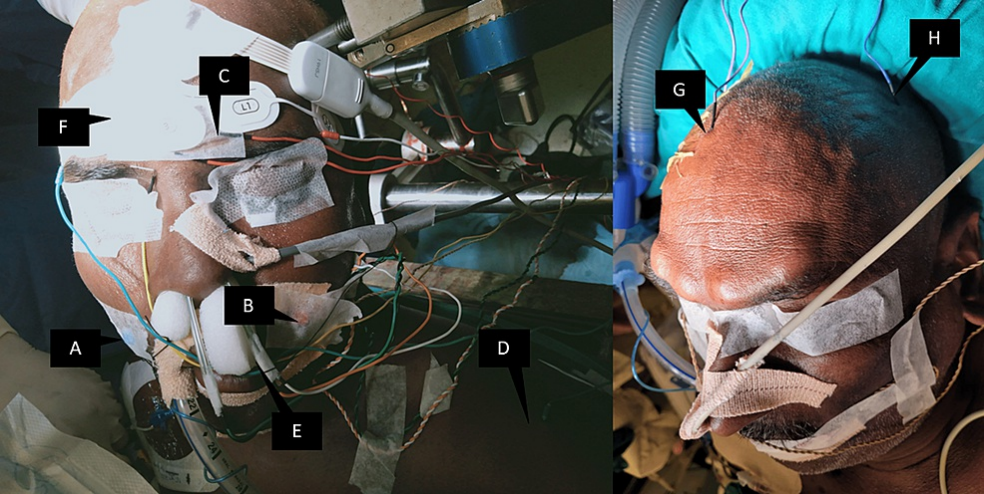
Two patients’ electrode positions are depicted. A: right upper lip electrode; B: left upper lip electrode; C: one of the supraorbital recording electrodes; D: ground electrode on the left shoulder; E: nasal cleft electrodes for olfactory evoked potentials; F: bispectral index strip for monitoring depth of anesthesia; G: Fz electrode; H: C5 electrode

We placed the stimulation electrodes (dual needle electrodes, Medtronics, Inc, Minneapolis, MN, USA) on one side at the upper lip and the other side on the lower lip. All electrode wires were isolated and secured from the adjacent cables and connected to the stimulation port of the monitoring device (NIM Eclipse, Medtronic Inc, Minneapolis, MN, USA). We placed recording electrodes at C5, C6, and Fz (using the 10-20 electroencephalography electrode placement system) and used a ground electrode on the masseter or A1 area or the shoulder (Figure 1).

We used various stimulation parameters but generally included settings of a rectangular pulse of current at a rate of 2.1-4.1 Hz, a pulse width of 200 microseconds, and a current intensity of 10-20 mA. We used a bandpass filter of 30-750 Hz. The sweep length was 100 ms, and we averaged 200 sweeps.

## Results

In this study, there were seven tumor cases, three aneurysms, one cavernoma, and one cystic lesion. All subjects underwent craniotomy procedures, including nine frontal, one temporal, and two parietal craniotomies. The mean age of the patients was 43 years, and 33% of them were males. The demographic data, diagnoses, electrode positions, and the outcomes of the various modalities we monitored are shown in Table 1.

**Table 1:** Demographics, diagnosis, procedures, and settings we used in the 12 patients. MAC: minimum alveolar concentration at the time of attempts to measure TSEP (we used inhalational agents at anesthesia induction in all patients except the 11th patient, in whom an almost total intravenous anesthesia regime was used); TSEP: trigeminal sensory evoked potential; MEP: motor evoked potential; OEP: olfactory evoked potential; SSEP: somatosensory evoked potential; VEP: visual evoked potential; +: obtained; -: not obtained despite monitoring; M: male; F: female; Rt SO: right supraorbital; Lf SO: left supraorbital

Case	Age	Sex	Diagnosis	Procedure	Stimulation point	Recording point	MAC	Current strength	Monitored modalities
1	32	M	Temporal glioma	Right frontotemporal craniotomy	Right upper lip, left lower lip	C5, C6, and Fpz	0.0	14–20 mA	TSEP (-)
2	24	M	Bilateral convexity meningioma	Biparietal craniotomy	Lower lips	Rt SO-FPz, Lf SO-Fpz, no C5, C6 electrodes	0.0	14–17 mA	MEP (+), TSEP (-), OEP(-)
3	34	F	Temporal cavernoma	Left temporal craniotomy	Both upper lips	C5, C6, and Fpz	0.3	14–17 mA	OEP (-), TSEP (-)
4	40	F	Parasagittal hydatid cystic lesion	Right parasagittal craniotomy	Right upper lip, left lower lip	C5, C6, Fpz* (1 cm above the eyebrow line)	0.0	14–17 mA	TSEP (-), OEP (-)
5	27	M	Temporoparital glioma	Frontotemporal flap removal temporal craniotomy	Left upper lip, right lower lip	C5, C6, and FPz	0.0	14–17 mA	MEP (+), TSEP (-), OEP (-)
6	43	F	Sphenoid wing meningioma	Right pterional craniotomy	Right upper lip, left lower lip	C5, C6, and Fpz	0.0	14–17 mA	MEP (+), SSEP (+), TSEP (-), OEP (-)
7	55	F	Anterior communicating artery aneurysm	Left frontal craniotomy	Both upper lips	C3, C4, and Fpz	0.2	14–20 mA	MEP (+), SSEP (+), TSEP (-), OEP (-)
8	50	F	Anterior communicating artery aneurysm	Left pterional craniotomy	Left upper lip, right lower lip	C3, C4, and Fpz	0.2	14–20 mA	MEP (+), SSEP (+), TSEP (-), OEP (-)
9	55	F	Posterior communicating artery aneurysm	Right frontal craniotomy	Right upper lip	C3, C4, and Cz	0.1	14–20 mA	MEP (+), SSEP (+), TSEP (-), OEP (-)
10	52	M	Temporal high-grade glioma	Left frontotemporal craniotomy	Right upper lip	C3, C4, and Cz	0.2	14–20 mA	TSEP (-), OEP (-)
11	34	F	Optic nerve glioma	Left frontal craniotomy	Right upper lip, left lower lip	C5, C6, and Fz area	0.0	14–17 mA	TSEP (+), VEP (+)
12	64	F	Parieto-occipital convexity meningioma	Left occipital craniotomy	Both lower lips	C5, C6, and Fz area	0.0	14–17 mA	TSEP (+)

**Figure 2:**
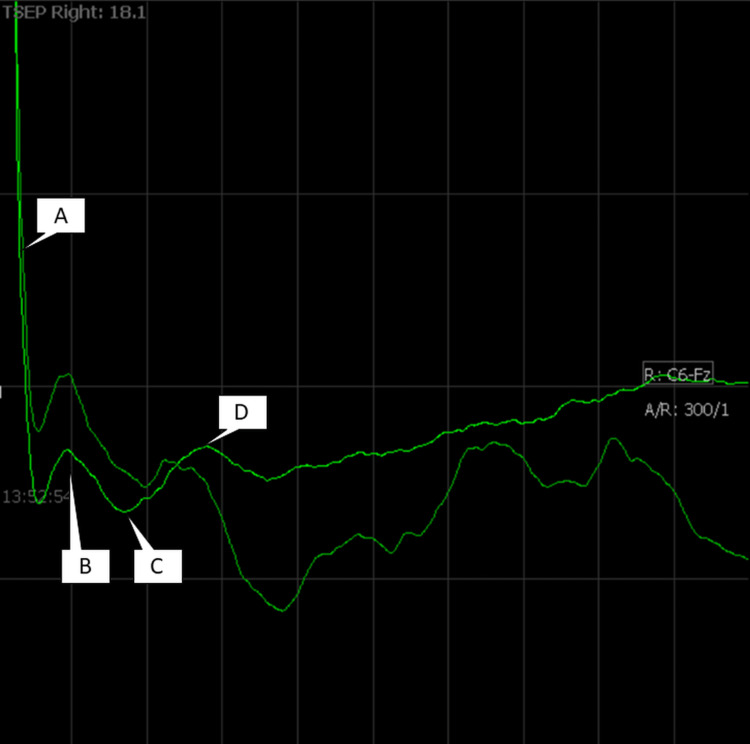
TSEP waveform in the 11th patient. The first negative wave (B) occurred at 10 milliseconds immediately after the large stimulus artifact (A). The first positive (downward) wave (C) was at 19-20 milliseconds, and the second negative wave (D) was seen at around 27 milliseconds. Two averages are shown in this figure. TSEP: trigeminal sensory evoked potential

After the patients were anesthetized, we started the monitoring immediately after positioning the patients on the headpins. We could obtain a reliable, reproducible, sustained waveform in only the last two of the 12 patients at 17 mA of current with the waves and latency of N13, P19, and N27 ms (Figure 2).

The waveforms started appearing only when the current reached a fairly high value of 15 mA starting from 5 mA, and it was quite prominent at 17 mA. The waveforms remained when we increased the stimulation rate from 1.7 Hz to 3.7 Hz. We did not use stimulation rates beyond 3.7 Hz in these two patients.

**Figure 3:**
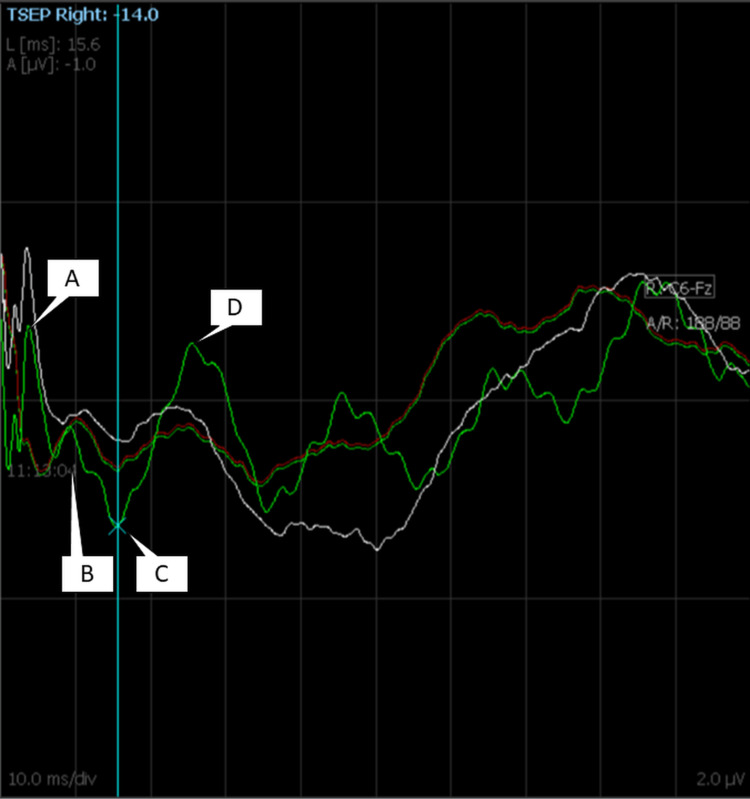
TSEP waveform in the 12th patient showing multiple averagings. Stimulus artifact (A) is followed by the first negative wave at 12 milliseconds, and then a positive wave is seen at 16 milliseconds. The second negative wave is seen at 27 milliseconds. An electrical line artifact is seen beyond the labeled areas in the green wave (last average) but not in others. The red wave is the baseline, and the white wave is the current running average. TSEP: trigeminal sensory evoked potential

The first negative peak that occurred after the stimulation was the clearest, and we could detect it in the two patients at 13 milliseconds. Sometimes, the latencies moved between 9 and 15 milliseconds during the procedure. The positive peak that occurred 20 ± 3 milliseconds after stimulation was frequently detected with greater amplitude than the other two waves. A second negative peak occurred at 27 ± 2 milliseconds. The waveforms were best seen before the skin incision (Figure 3).

We used a relatively lower pulse duration of 50-150 microseconds and a lower rate of stimulation, usually between 1.3 Hz and 2.7 Hz at a threshold intensity of 14 mA to 17 mA in the last two patients compared to higher rates and pulse widths in the earlier patients. The bandpass filter was 5 Hz to 750 Hz. When we used a notch filter, it produced a large artifact that reversed in polarity when we inversed the stimulus polarity. Hence, we avoided notch filters to prevent the artifact from eclipsing the waveforms. The true waveform we obtained in the last two patients was constant and did not reverse in polarity on inversing the stimulus polarities. We completely turned off the inhalational agents in the patients from whom we eventually obtained responses. Both these patients were also under muscle relaxants. The second patient we got the response for was operated on in the sitting position.

Using cautery and other instruments such as power drills almost invariably caused waveform distortions, but they tended to become relatively more stable after the craniotomy.

## Discussion

Although SSEPs are now a well-established modality both in the laboratory and operation rooms, TSEP recordings have been difficult to achieve during surgeries due to various factors. However, it has a role in an awake patient in the neurophysiology laboratory to distinguish between primary and secondary trigeminal neuralgia. To our knowledge, long-latency TSEP has been demonstrated only once under general anesthesia [8].

TSEP waveforms are called short and long latency, depending upon whether the recognizable waves occur before or after 10 milliseconds [9]. The origin of short-latency responses is controversial, and some researchers now consider it an artifact of muscle activity [7]. Various researchers have reported sequences of positive and negative waves at multiple latencies. Stechison and Kralick concluded in 1993 that they have no practical value, after eliciting long-latency TSEP in awake patients but failing to generate them when those patients were anesthetized [9]. In awake patients, they found out that the lower lips were most efficient in generating the waveforms compared to five other sites, including the mental nerve. However, Stechison could elicit short-latency TSEP from the extra-axial trigeminal system showing a potential to monitor that part of the system during surgery [10]. Only later, in 2011, Malcharek et al. reported the successful acquisition of the TSEP under general anesthesia in 99 patients by completely avoiding inhalational agents. They also found that using muscle relaxants improved the independence of the recordings [8]. We used only lower lip stimulation in the last patient in whom we got a good response and used muscle relaxants in both patients because we were not monitoring motor evoked potentials simultaneously.

Our study findings indicate that an inhalational agent, even when used at low doses, might obliterate or reduce TSEP. In the 11th patient, we used an almost total intravenous anesthesia regime. The last patient, too, in whom we got the responses, was an instance where a very low concentration of isoflurane was initially used, but it was turned off later. We did not use nitrous oxide in any of the patients. Xiang et al., in a randomized controlled trial, confirmed that escalating the minimum alveolar concentrations of desflurane and sevoflurane by 0.3, 0.6, and 0.9 reduced the amplitudes and increased the latencies of both SSEPs and motor evoked potentials during neurosurgery [11]. It appears that the TSEP is also particularly vulnerable to the use of inhalational agents.

Limitations of the study

Our study included only a relatively few patients but the waveforms in the last two patients were stable enough to be convincingly described as TSEP. We did not increase the inhalational anesthetics to examine at what point the waveforms disappeared in these two patients. We concluded that the higher levels of these agents used in the other patients prevented us from eliciting the responses. However, it is possible that minor changes in technique rather than inhalational agents were responsible for negative results in the first 10 patients.

## Conclusions

We could elicit long-latency TSEP in a minority of neurosurgical patients, even with restricted use of inhalation agents at induction. We electrically stimulated the lips with a relatively lower rate and duration, which appeared to facilitate the response. We think that total intravenous anesthesia or turning off the inhalational agents early during routine general anesthesia is required to elicit the response. The responses are most clear before the skin incision. They remained relatively stable once the craniotomy was over when the interference from using other equipment, such as monopolar cautery, became minimal. One should avoid using a notch filter to prevent an artifactual response from eclipsing the true TSEP waveforms.
